# Feasible Relation between Glutathione Peroxidase and Febrile Seizure

**Published:** 2017

**Authors:** Abolfazl MAHYAR, Parviz AYAZI, Reza DALIRANI, Behzad MOHAMMAD HOSEINI, Mohammad Reza SAROOKHANI, Amir JAVADI, Shiva ESMAEILY

**Affiliations:** 1 Department of Pediatrics, Qazvin Children hospital, Qazvin University of Medical Sciences, Qazvin, Iran; 2 Pediatric Nephrology Department Mofid children hospital, Shahid Beheshti University of Medical Sciences, Tehran, Iran; 3 Cell and Molecular Research Center, Qazvin University of Medical Sciences, Qazvin, Iran; 4 Department of Statistics, Qazvin University of Medical Sciences, Qazvin, Iran

**Keywords:** Febrile Seizure, Glutathione Peroxidase

## Abstract

**Objective:**

We aimed to determine the relationship between serum glutathione peroxidase and febrile seizure.

**Materials & Methods:**

In this case-control study, 43 children with simple febrile seizure (case group) were compared with 43 febrile children without seizure (control group) in terms of serum glutathione peroxidase level, measured by ELISA method. This study was conducted in Qazvin Children Hospital, Qazvin University of Medical Sciences in Qazvin, Iran in 2012-2013. The results were analyzed and compared in two groups**.**

**Results:**

From 43 children 24 (53%) were male and 19 (47%) were female in children with simple febrile seizure, and 26 (60%) were male and 17 (40%) were female in febrile children without seizure (control group) (P=0.827). Serum glutathione peroxidase level was 166 U/ml (SD=107) in the case group and 141 U/ml (SD=90.5) in the control group of no significant difference.

**Conclusion:**

There was no significant relationship between serum glutathione peroxidase and simple febrile seizure. Thus, it seems that glutathione peroxidase, an essential component of antioxidant system, does not play any role in the pathogenesis of simple febrile seizure.

## Introduction

Febrile seizure is the most common type of seizure in children. This type of seizure is common at the age of 6 months to 5 yr developed following a fever over 38 °C. In this type of seizure, there is no central nervous system infection, metabolic diseases, and electrolyte disturbances. The prevalence of disease varies between 3.4-9.3% (1-6). The disease is divided into simple and complex types. The former, lasts less than 15 min, does not repeat within 24 h and is generalized. In contrast, the latter is partial seizure, lasts more than 15 minutes and repeats within 24 hours. Although prognosis of febrile seizure is generally good, there is a risk of epilepsy after prolonged complex seizure (7). 

Although several studies showed the relationship between micronutrients such as iron, zinc and cytokines and febrile seizure, the exact etiology of the disease is unknown (8, 9). Oxidative stress and production of reactive oxygen species (ROS) are cause and effect of seizure attacks. In addition, antioxidants deficiency is a risk factor for seizure attacks (10, 11). Body’s antioxidant system consists of antioxidant enzymes such as glutathione peroxidase (GPx), minerals such as selenium and vitamins such as vitamin A (12). GPx is the most important antioxidant enzyme in the body and protects body from oxidative damages. Biochemical function of the enzyme is regeneration of lipid hydroperoxide and hydrogen peroxide (13). 

Given the role of oxidative stress in seizure (14, 15), this study was conducted to determine the relationship between serum GPx and simple febrile seizure in children.

## Materials & Methods

In this case-control study, 43 children with simple febrile seizure (case group) were compared with 43 febrile children without seizure (control group) in terms of serum GPx levels. This study was conducted in Qazvin Children Hospital, Qazvin University of Medical Sciences in Qazvin, Iran in 2012-2013. The age of children was between 6 months and 5 yr. The sample size was calculated according to Mean0 =440.57 nmol/min/ml, SD0=264.00 Mean1 =801.00 nmol/min/ ml, SD1 =267.00 to provide 95% confidence coefficient and 90% power in statistical analysis (10). 

Consecutive sampling was used until the required sample size was obtained. Inclusion criteria for the case group were 1) seizure resulting from a fever over 38 °C; 2) generalized seizure; 3) seizure less than 15 min and 4) the occurrence of a seizure in 24 h without repetition) (2, 3). Children with complex febrile seizure, epilepsy, central nervous system infection (such as meningitis, encephalitis or any other infections), electrolyte disturbances such as hyponatremia or hypernatremia, metabolic diseases such as hypocalcemia and inborn error diseases, central nervous system abnormalities such as hydrocephaly and any other factors justifying patient’s seizure were excluded . 

The control group was selected by group matching method from febrile children without seizure attending the hospital due to simple infections such as common cold that required no special medication. Both groups were from and lived in Qazvin and there was no seizure history in the family. The two groups were matched for age, gender, weight, and height and head circumference. The temperature was measured with a mercury thermometer in the armpit, and the weight, height and head circumference were measured by standard methods (3). 

First, a questionnaire was developed and then the plan was explained to parents. After parent’s willingness to participate in the project and the approval of the Ethics Committee of the Research Department of Qazvin University of Medical Sciences, informed consent form was signed by parents. 

A sample of 3 ml venous blood was taken from children. Serums of blood samples were separated after centrifugation and kept in -20 °C. Considering the compensatory elevation of the GPx activity in the brain tissue within a few days following the seizure attack (Li-Ping and Patel, 2006), the patients were expected to be seizure-free for at least 2 weeks before sampling to let the GPx activity return to the baseline level (10, 14, 15). Serum GPx was measured by ELISA method with gluthathione peroxidase (GSH-PX) -Humman-ELISA 96t- Glory kit (Glory Science Company, the U.S.A, Lot No. 20130123) in Faculty of Paramedicine of Qazvin University of Medical Sciences with ELISA reader (Lab System MS, Finland). 

Data were analyzed by chi-square test and t-test using SPSS version 16 (Chicago, IL, USA). P value<0.05 was considered significant. 

## Results

From 43 children with simple febrile seizure (case group), 24 children (53%) were male and 19 children (47%) female. These values in children without seizure (control group) were 26 (60%) and 17 (40%), respectively (P=0.827). The mean age in case and control groups were 20.1±11.7 and 20.5±13 months, respectively (P=0.89). There was no significant difference between the two groups in terms of gender, age, weight, height, head circumference and temperature (P>0.05) (Table 1). 

The minimum, maximum and mean±SD of serum GPx level in the case group were 44, 442 and 166±107 U/ ml. These values in control group were 35, 417 and 141±90.5U/ml. No significant difference was observed between the two groups in terms of serum GPx levels (P=0.244) (Table 2).

## Discussion

This study showed that there was no significant difference between children with simple febrile seizure and healthy children in terms of serum GPx levels. Febrile seizure is the most common type of seizure in children and despite numerous studies conducted, the pathogenesis of this disease is still unknown. Given the role of GPx in epilepsy, this question was raised for us on the status of serum GPx activity in patients with febrile seizure. 

**Table 1:** Comparison of Variables Between Case and Control Group

**Variables**	**Case group**	**Control group**	***P***
**Male/Female** 1	24/19	26/17	0.82
**Age (month) ** 2	20.1±11.7	20.5±13	0.89
**Weight (kg) ** 2	11.1±2.1	10.2±2	0.062
**Height (cm) ** 2	80.5±11.4	80.6±10	0.94
**Head circumference (cm) ** 2	46.5±3.1	45.9±2.5	0.36
**Temperature (0c) ** 2	38.6±0.6	38.4±0.4	0.056

1 Chi- square test,

2 T- Test (Mean ±SD)

**Table 2:** Comparison of Serum Glutathione Peroxidase Level in Case and Control Group

**Glutathione peroxidase** **(U/ml)** 1	**Case group**	**Control group**	***P***
**Mean±SD**	166±107	141±90.5	0.24
**Minimum**	44	35	
**Maximum**	442	417	

1 T- Test (Mean ±SD)

This study is the first one that investigated serum GPx levels in children with febrile seizure. However, several studies have noted the relationship between serum GPx and epilepsy, with controversial results (10, 16-20). The study of Ashrafi et al. on 53 children with epilepsy and 57 healthy children showed that serum selenium, GPx activity in red blood cells and serum selenium in children with epilepsy were less than those of the control group. No significant relationship was found (10). In four patients with refractory seizure, the intracellular glutathione peroxidase levels were lower than normal (16). In two patients, serum selenium level was normal, plasma GPx level was increased, and intracellular GPx activity was lower than normal. In contrast, in two other patients, serum selenium levels and plasma GPx levels were low. Patients’ seizure attacks were discontinued by discontinuation of anti-seizure medication and selenium administration (16). Lack of GPx was involved in the pathogenesis of epilepsy (10, 16). A study on 29 patients with epilepsy and 50 healthy subjects aged 20-50 yr showed that plasma GPx levels in patients with epilepsy before and after treatment with anti-seizure medication was higher than the control group. The authors suggest that high levels of plasma GPx can be attributed to increased concentrations of H2O2 in RBC (17). 

In contrast, there are reports indicating that GPx activity levels in patients with epilepsy do not change (18-20). A study on 36 patients aged 14-18 yr and 40 healthy subjects demonstrated that no significant difference was observed between plasma GPx and red blood cells of children with epilepsy and the control group (18). The authors believe that GPx does not contribute to the pathogenesis of epilepsy in children. The results of present study are consistent with previous studies (19, 20). Antioxidant role of GPx is emphasized earlier (21, 22). Following biologic redox reactions, free radicals such as ROS (radical superoxide, hydrogen peroxide, and hydroxyl) and nitrogen species are generated. These materials are highly toxic for the body, especially the brain. Failure in ROS detoxification by enzymatic and non-enzymatic antioxidants such as GPx increases the risk of severe oxidative damages in brain cells (21, 22). 

GPx is one of the most important antioxidant enzymes in humans. This enzyme is a tetrameric glycoprotein containing selenium. Each of its molecules contains four selenium atoms. So far, 8 types of GPx (GPx1- GPx8) have been identified and GPx1 is the most common type (13, 23). Studies on the relationship between GPx and selenium are contradictory. Although some researchers have noted the relationship between GPx activity and selenium levels, others do not believe (10, 16, 24). Researchers who believe there is a direct relationship between GPx activity and selenium suggest that instead of measuring the level of selenium that is difficult and costly, GPx is used (24, 25). According our findings the GPx is not a suitable substitute for selenium measurement in febrile seizure patients.

 There are some limitations of this study including not measuring GPx in CSF of our simple febrile patients and children with complex febrile seizure and not simultaneous measurement of serum selenium levels due to limited facilities. Further studies are recommended in this area.

**In conclusion,** it seems that glutathione peroxidase, does not play any role in the pathogenesis of simple febrile seizure.

## Authors’ contribution

Abolfazl Mahyar ,Parviz Ayazi , Reza Dalirani :Project Design and supervise, Behzad Mohammad Hoseini; Data collection, Mohammad Reza Sarookhani; Perform of laboratory test, Amir Javadi , Shiva Esmaeily; Statistical calculation and analysis. All authors agreed to be accountable for all aspects of the work in ensuring that questions related to the accuracy or integrity of any part of the work are appropriately investigated and resolved. 
